# Experimental infection of highly pathogenic avian influenza virus H5N1 in black-headed gulls (*Chroicocephalus ridibundus*)

**DOI:** 10.1186/s13567-014-0084-9

**Published:** 2014-08-19

**Authors:** Antonio Ramis, Geert van Amerongen, Marco van de Bildt, Loneke Leijten, Raphael Vanderstichel, Albert Osterhaus, Thijs Kuiken

**Affiliations:** CReSA and Departament de Sanitat i Anatomia Animals, Universitat Autonoma de Barcelona, Barcelona, Spain; Department of Viroscience, Erasmus Medical Center, Rotterdam, The Netherlands; Department of Health Management, Atlantic Veterinary College, University of Prince Edward Island, Charlottetown, Canada

## Abstract

Historically, highly pathogenic avian influenza viruses (HPAIV) rarely resulted in infection or clinical disease in wild birds. However, since 2002, disease and mortality from natural HPAIV H5N1 infection have been observed in wild birds including gulls. We performed an experimental HPAIV H5N1 infection of black-headed gulls (*Chroicocephalus ridibundus*) to determine their susceptibility to infection and disease from this virus, pattern of viral shedding, clinical signs, pathological changes and viral tissue distribution. We inoculated sixteen black-headed gulls with 1 × 10^4^ median tissue culture infectious dose HPAIV H5N1 (A/turkey/Turkey/1/2005) intratracheally and intraesophageally. Birds were monitored daily until 12 days post inoculation (dpi). Oropharyngeal and cloacal swabs were collected daily to detect viral shedding. Necropsies from birds were performed at 2, 4, 5, 6, 7, and 12 dpi. Sampling from selected tissues was done for histopathology, immunohistochemical detection of viral antigen, PCR, and viral isolation. Our study shows that all inoculated birds were productively infected, developed systemic disease, and had a high morbidity and mortality rate. Virus was detected mainly in the respiratory tract on the first days after inoculation, and then concentrated more in pancreas and central nervous system from 4 dpi onwards. Birds shed infectious virus until 7 dpi from the pharynx and 6 dpi from the cloaca. We conclude that black-headed gulls are highly susceptible to disease with a high mortality rate and are thus more likely to act as sentinel species for the presence of the virus than as long-distance carriers of the virus to new geographical areas.

**Electronic supplementary material:**

The online version of this article (doi:10.1186/s13567-014-0084-9) contains supplementary material, which is available to authorized users.

## Introduction

Historically, highly pathogenic avian influenza viruses (HPAIV) were rarely found in wild birds, and, if they were, they typically did not cause clinical disease [1]. However, in late 2002, HPAIV H5N1 outbreaks were described in Hong Kong parks causing disease and death in wild local and migratory birds; concurrently with these outbreaks, HPAIV H5N1 were isolated from dead chickens and human beings [2]. It was shown through antigenic analysis that these new isolates of HPAIV H5N1 presented a reactivity pattern different from that of HPAIV H5N1 isolated in 1997 and 2001 and caused systemic infection in experimentally infected domestic ducks, with high virus titres and lesions in multiple organs, particularly in the brain [3].

Since 2002, disease and mortality from natural HPAIV H5N1 infection have been described for several gull species: black-headed gull (*Chroicocephalus ridibundus*) [2], great black-headed gull (*Larus ichthyaetus*) and brown-headed gull (*Larus brunnicephalus*) [4]. Also, experimental infections have been performed in laughing gulls (*Larus atricilla*) [5] and herring gulls (*Larus argentatus*) [6] showing that these species were highly susceptible (100%) to different HPAIV H5N1 isolates (A/WhooperSwan/Mongolia/244/05 and A/Duck Meat/Anyang/01), presenting a severe and short (4 to 10 dpi) clinical course, consisting mainly of neurologic signs with a high mortality rate (up to 100% in some cases). The main target organs for viral replication were central nervous system (CNS), pancreas, and adrenal gland, and the duration of the viral shedding after inoculation was in a range of 1 to 10 dpi depending on the isolate, the gull species, and inoculation route. In these experiments, virus dynamics and in-depth pathogenesis were not investigated. Lastly, in an experimental infection in common gulls (*Larus canus*) with a HPAIV H5N1 isolate (A/Gull/Chany/P/06) isolated from healthy birds of this species, the virus was shown to replicate in respiratory and digestive tracts and to be excreted from pharynx and cloaca for 2 weeks after the infection, with no clinical signs in infected birds [7].

The black-headed gull is the most ubiquitous gull in Europe and Asia, is a peridomestic species that often occurs in urban and agricultural environments [8] and is known to be susceptible to HPAIV H5N1 infection [2]. Also, black-headed gulls are listed by the European Food Safety Authority [9] as having higher probability to be exposed to Asian linage HPAIV H5N1 during migration outside the European Union, because of their gregarious behaviour and their tendency to use both wild areas and built-up areas. However, it is known neither how long black-headed gulls can shed HPAIV H5N1 nor how susceptible they are to disease from HPAIV H5N1 infection. These are important considerations to decide whether black-headed gulls are more likely to act as sentinels for the presence of the virus in the environment, or to act as long-distance vectors of the virus to other geographical areas [10]. Therefore, we performed an experimental infection of black-headed gulls using HPAIV A/turkey/Turkey/1/2005 (H5N1) to determine the susceptibility of this host species to infection and disease from this virus; the pattern of pharyngeal and cloacal viral shedding; the clinical signs and pathological changes; and lastly the virus distribution in different tissues. We chose this virus isolate to allow comparison of the results between black-headed gulls and 6 different wild species of Anseriformes [10], which jointly with Charadriiformes—to which the black-headed gull belongs—have traditionally been considered the natural reservoir for avian influenza viruses; and also because this isolate had spread quickly from Asia to Europe in 2005–2006 [11].

## Material and methods

### Virus preparation

A virus stock of HPAIV A/turkey/Turkey 1/2005 (H5N1) was prepared by propagation in Madin-Darby canine kidney (MDCK) cells and titrated according to standard methods [12]. The fluid had a titre of 1 × 10^8.1^ median tissue culture infectious dose (TCID_50_)/mL and was diluted with phosphate-buffered saline (PBS) to obtain a final titre of 10^4^ TCID_50_/mL. All the experiments with HPAIV H5N1 were performed under Biosafety level 3 + conditions.

### Experimental design

We used 22 black-headed gulls (2–3 weeks old) obtained from a breeding colony site at Blauwestad, The Netherlands. The gulls were housed in negative isolator units for 15 days to acclimatize them to the new environment. Two days before the experimental inoculation, they were tested for antibodies against influenza viruses by use of a commercially available nucleoprotein-based ELISA test (European Veterinary Laboratory, Woerden, The Netherlands) to assure they were not previously infected. Sixteen birds were inoculated with 1 × 10^4^ TCID_50_ HPAIV H5N1, 1.5 mL intratracheally and 1.5 mL intraoesophageally, which was the same virus, inoculum dose, and inoculation route as in a previous study [10]. We used this dose to increase the chance of inducing subclinical infection and to simulate field circumstances. In addition, 6 birds that served as negative controls were sham-inoculated in the same manner with PBS-diluted sterile allantoic fluid. Daily, a qualified veterinarian scored clinical signs of disease in all birds according to a standardized list. Also daily, the body weight of each bird was measured, and cloacal and pharyngeal swabs were collected in 1 mL transport medium. Birds were euthanatized by exsanguination under isofluorane anesthesia. After euthanasia, blood samples were taken and necropsies and tissue sampling were performed. The original plan was to euthanatize four HPAIV H5N1-inoculated birds on each of days 2, 4, 6 and 12 post inoculation, two sham-inoculated birds at 3 days post inoculation (dpi) and four sham-inoculated birds at 12 dpi. Because some H5N1-inoculated birds died spontaneously before the planned day of euthanasia, we were unable to completely follow the original plan, and the timing of necropsies and sampling for H5N1-inoculated birds was as follows: four birds at 2 dpi; five birds at 4 dpi; two birds at 5 dpi; two birds at 6 dpi; one bird at 7 dpi; and two birds at day 12 dpi (Table 1).

**Table 1:** **Follow up of infected gulls**

	**Number of birds per day**											
**Day post inoculation**	**1 dpi**	**2 dpi**	**3 dpi**	**4 dpi**	**5 dpi**	**6 dpi**	**7 dpi**	**8 dpi**	**9 dpi**	**10 dpi**	**11 dpi**	**12 dpi**
**Total alive**	16	16	12	12	7	5	3	2	2	2	2	2
**With neurologic signs**	0	0	1	2	1	3	2	2	2	2	2	2
**Died spontaneously**	0	0	0	1	2	0	1	0	0	0	0	0
**Euthanatized according to programme**	0	4	0	4	0	2	0	0	0	0	0	2
**Total necropsied**	0	4	0	5	2	2	1	0	0	0	0	2

Number of birds showing neurological signs, spontaneous deaths, and programmed euthanasias in H5N1-inoculated black-headed gulls during the course of the experiment.

### Pathologic and immunohistologic examination

Necropsies and tissue sampling were performed according to a standard protocol. After fixation in 10% neutral-buffered formalin and embedding in paraffin, 3-μm-thick tissue sections were made and stained by two methods: hematoxylin and eosin staining for histologic evaluation and an immunohistologic method that used a monoclonal antibody against nucleoprotein for influenza A virus as a primary antibody for detection of influenza viral antigen [12]. The positive control was a lung of a HPAIV (H5N1) infected ferret; negative controls were substitution of the primary antibody by an irrelevant monoclonal antibody of the same isotype, and testing of tissues from sham-inoculated black-headed gulls. The following tissues were examined: brain (cerebrum, cerebellum and brain stem), trachea, bronchus, lung, air sac, oesophagus, proventriculus, duodenum, pancreas, jejunum, ileum, cecum, colon, liver, gallbladder, cloaca, cloacal bursa, spleen, thymus, kidney (cranial and caudal lobes), gonad, adrenal gland, feathered skin, myocardium and skeletal muscle.

### Virus quantitation by real time RT-PCR

Total nucleic acids were isolated on a MagnaPure LC system using the LC Total Nucleic Acid Isolation Kit (Roche Diagnostics, Almere, The Netherlands) according to the manufacturer’s protocol. Influenza A virus was detected using a real-time PCR assay [13]. Amplification and detection were performed on an ABI7500 (Applied Biosystems, Foster City, CA, USA) with the Taqman Fast Virus 1-Step Master Mix.

### Virus titration

Tissue samples were weighed and homogenized in 1 mL of transport medium with a FastPrep-24 homogenizer (MP Biomedicals, Eindhoven, The Netherlands). Virus titres were determined by serial 10-fold dilution of the homogenized tissue samples and swabs on MDCK cells as described previously [14].

### Statistics

The area under the curve for viral loads from pharyngeal and cloacal swabs within gulls, as measured by RT-PCR (Ct value) and virus isolation (10^x^ TCID_50_), were compared using the Wilcoxon matched-pairs signed-ranks test [15]. A multilevel mixed-effects linear regression, with gull weight (g) as the dependent variable, was fit using maximum likelihood estimation, and included a random effect for gull and first order auto-regressive (AR1) structured residual errors between repeated weight measurements. A fractional polynomial [16], using two terms, was used to estimate weight over time. Normality and homoscedasticity of residuals, at both levels of the model, were evaluated [17], and statistical significance was set at *P* < 0.05.

## Results

### Virus detection in pharyngeal and cloacal swabs

**Figure 1:**
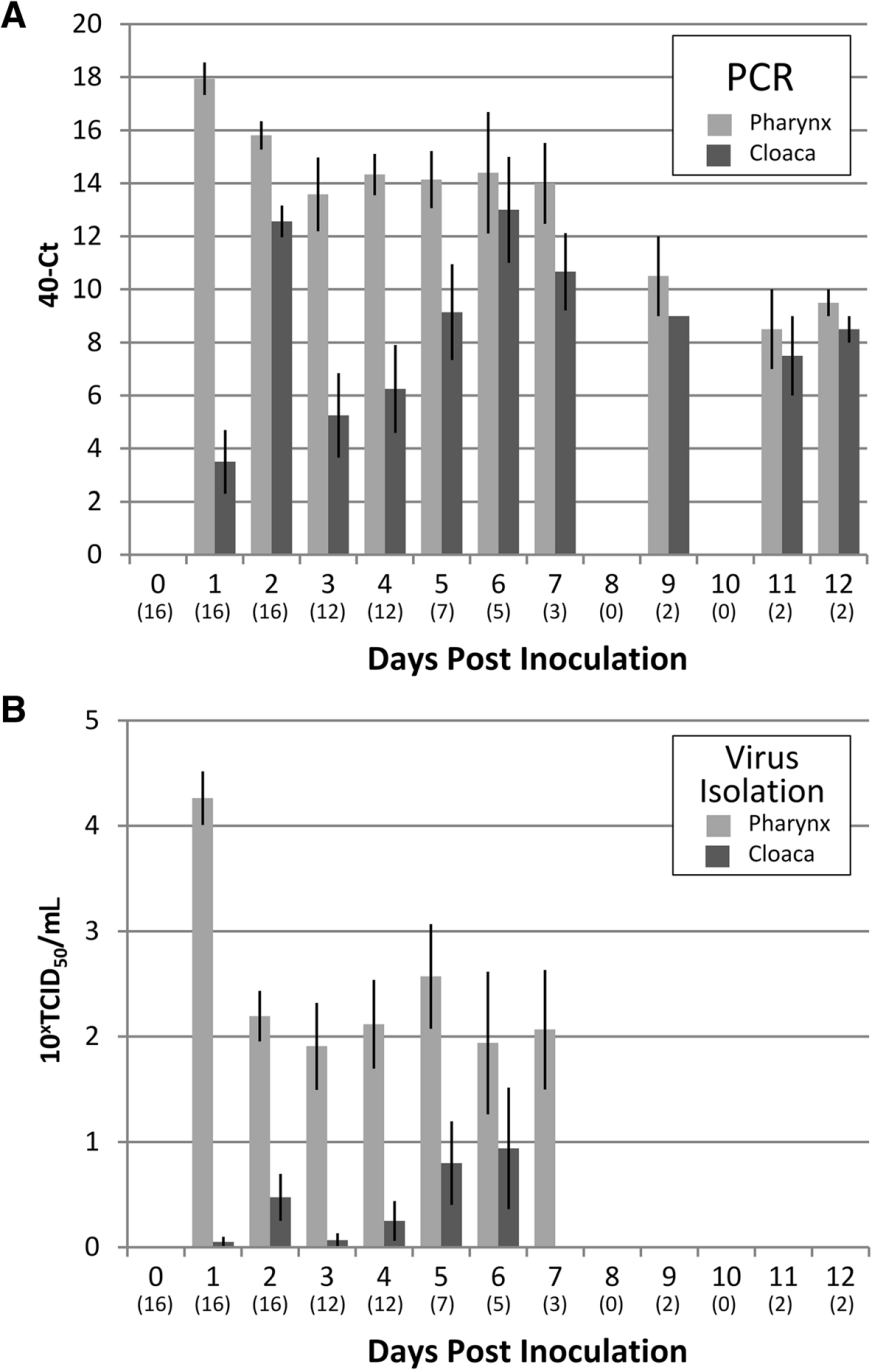
**Viral detection in pharyngeal and cloacal swabs using RT-PCR and virus isolation.** Bar graph with standard error estimates of results of viral detection in pharyngeal and cloacal swabs taken from gulls inoculated on day 0 with HPAIV H5N1 virus and followed for 12 days. **(A)** Real-time RT-PCR, expressed as 40 (minimum cycling threshold [Ct] value considered negative) – actual Ct value. **(B)** Virus isolation, expressed as 10^x^ × median tissue culture infectious dose per mL (TCID_50_/mL), with a titre < 10^0.8^ TCID_50_/mL considered negative. Numbers below each day (in parentheses) represent total number of birds tested on that day. Each bar represents the average value for all birds tested on that day.

Virus was detected in the pharyngeal swabs, cloacal swabs, or both, of all 16 H5N1-inoculated birds, indicating that they were all productively infected (Table 2). Based on the area under the curve, there were larger quantities of virus detected in pharyngeal swabs than in cloacal swabs using RT-PCR and virus isolation (*P <* 0.001) (Figure 1A, B). By RT-PCR, nearly all pharyngeal swabs tested positive from 1 dpi to the end of the experiment at 12 dpi. The mean Ct-value was highest at 1 dpi (Ct value 22) and declined slowly on subsequent days. Virus was isolated from RT-PCR-positive pharyngeal swabs from most birds from 1 to 7 dpi, but could no longer be isolated on subsequent days. The mean virus titre in pharyngeal swabs was highest (10^4.26^ TCID_50_) at 1 dpi and between about 10^2^ and 10^3^ TCID_50_ from 2 to 7 dpi. By RT-PCR, most cloacal swabs tested positive from 1 dpi to the end of the experiment at 12 dpi, but the Ct values were generally higher (i.e. less virus) than in pharyngeal swabs. The mean Ct-value slowly decreased until 6 dpi (Ct value 27) and slowly increased on subsequent days. Virus was isolated only from a small proportion of RT-PCR-positive cloacal swabs from 1 to 6 dpi, with mean virus titres between detection limit and just above 10^2^ TCID_50_, and could not be isolated on subsequent days. No virus was detected by RT-PCR in any pharyngeal or cloacal swabs of sham-inoculated birds.

**Table 2:** **Virus detection in pharyngeal and cloacal swabs of H5N1-inoculated gulls during the course of the experiment**

		**Pharyngeal swab**				**Cloacal swab**			
		**RT-PCR**		**Virus isolation**		**RT-PCR**		**Virus isolation**	
**Day**	**No. tested**	**No. positive**	**Ct range**	No. positive*	**Titre range**	**No. positive**	**Ct range**	No. positive*	**Titre range**
**1**	16	16	19-27	16	1.5-5.5	6	27-32	1	< 0.8-0.8
**2**	16	16	21-29	15	1.2-3.5	16	22-30	4	1.5-2.8
**3**	12	11	21-29	10	0.8-4.2	6	28-31	1	< 0.8-0.8
**4**	12	12	22-30	9	1.8-3.8	7	26-33	2	0.8-2.2
**5**	7	7	23-32	6	1.8-3.8	6	24-31	3	1.2-2.2
**6**	5	5	20-34	4	0.8-3.5	5	20-30	2	2.2-2.5
**7**	3	3	23-28	3	1.5-3.2	3	27-32	0	n.a.
**9**	2	2	31	0	n.a.	2	28-31	0	n.a.
**11**	2	2	30-33	0	n.a.	2	31-34	0	n.a.
**12**	2	2	30-31	0	n.a.	2	31-32	0	n.a.

Virus isolation was attempted on all PCR-positive samples.

n.a., not applicable.

### Clinical signs

**Figure 2:**
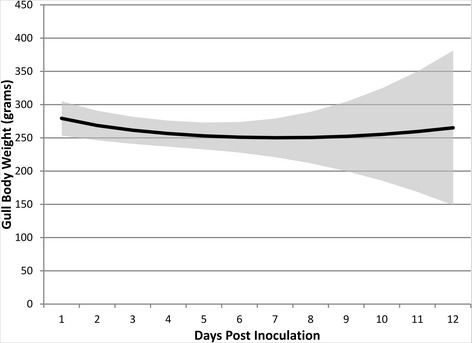
**Estimated gull body weights (g) throughout the study period.** It was determined by the fractional polynomial terms in the mixed-effects linear regression, for control gulls that remained alive for the duration of the study; shaded area represents the 95% confidence intervals. The same statistical model predicted an average constant loss of 26 grams for H5N1-inoculated gulls (*P* = 0.029), and an average constant loss of 26 grams for gulls that died during the study (*P* = 0.047)—these estimates are not shown in graph.

During the first 3 dpi, no clinical signs were observed in H5N1-inoculated birds, except one bird (#49) that showed less activity than normal and slight nonspecific neurological signs at 3 dpi: regularly fluffing of feathers and walking slightly to one side. At 4, 5 and 6 dpi, all H5N1-inoculated birds were less active than sham-inoculated birds, showing increased recumbency and regularly fluffing of feathers. Four of them (#46, 47, 49, 51) showed obvious neurological signs, consisting mainly of torticolis, circling, loss of balance and head tremors. Respiratory or digestive signs, such as diarrhoea, were not observed in any H5N1-inoculated birds. From 7 dpi onwards, the two remaining H5N1-inoculated birds (#47, 51) partially recovered, alternating between normality and neurological signs (Table 1). At 4 dpi, the H5N1-inoculated bird that showed clinical signs at 3 dpi (#49) died spontaneously. Two birds (#46, 50) at 5 dpi and one bird (#48) at 7 dpi, all three H5N1-inoculated, also died spontaneously. No clinical signs or spontaneous mortality were observed in any of the sham-inoculated birds. Based on the statistical model: the body weight of H5N1-inoculated birds was 26 g lower than sham-inoculated birds (*P* = 0.029) after inoculations; within the H5N1-inoculated group, the body weight of birds that died was 26 g lower after inoculations than that of birds that survived (*P* = 0.047). Regardless of their inoculation status, gulls were losing weight during the first week post inoculation, and this trend was reversed after 10 dpi (*P* < 0.001, Figure 2).

### Gross lesions

No relevant gross lesions were observed in any H5N1-inoculated or sham-inoculated birds. In general, all birds showed good body condition and had abundant subcutaneous and perivisceral fat deposits.

### Histopathology and immunohistochemistry

**Figure 3:**
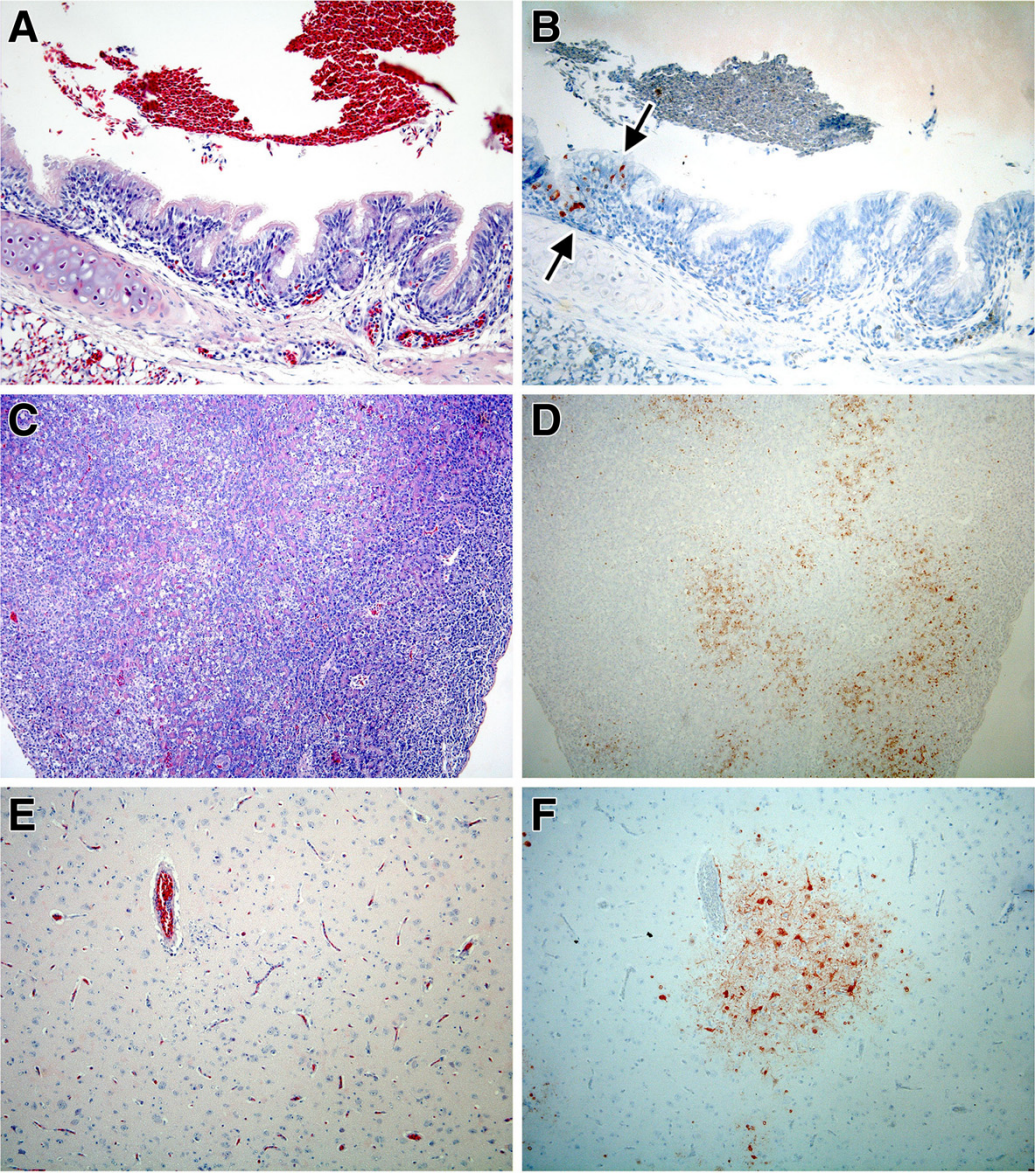
**Histopathology and antigen expression in selected tissues from infected gulls.** By immunohistochemistry, influenza virus antigen expression is visible as a red staining. **(A, B)**: Bronchus of 2 dpi infected gull without histologic lesions **(A)**, and scarce bronchial epithelial cells showing positivity on the immuno-stained consecutive section (arrows) **(B)**. **(C, D)**: Pancreas of 4 dpi infected gull showing multiple foci of lytic necrosis **(C)**, and intense positivity associate to necrotic foci in the immune-stained consecutive section. **(E, F)**: Cerebral cortex showing necrotic neurons, and satellitosis **(E)**, and intense positivity in neurons and associated glial cells in the immune-stained consecutive section. HE stain **(A, C, E)** and immunoperoxidase counterstained with hematoxylin **(B, D, F)**. All the pictures are at 10x power fields.

The evaluation of histological lesions and the presence of virus antigen in the different cells and tissues of evaluated organs of H5N1-inoculated birds are summarized in Table 3. On day 2, air sacs in all birds (#36, 37, 38, 39) had marked diffuse infiltration of heterophils and multiple variably-sized well-demarcated aggregates of heterophils and macrophages. The adrenal gland in one bird (#37) had multiple foci of necrotic adrenocortical and chromaffin cells. Virus antigen expression was observed in epithelial cells (#36, 37, 38, 39) and unspecified inflammatory cells (#36, 37, 38, 39) of the air sacs, and in adrenocortical cells (#37) of the adrenal gland, localized to lesions in these tissues. Virus antigen expression also was observed in individual tracheal and bronchial ciliated epithelial cells (#38) (Figure 3A, B), air capillary cells (#36, 37), and thymic epithelial cells in absence of histological lesions.

**Table 3:** **Histological lesions (HE) and presence of antigen (IHC) in tissues of H5N1-inoculated gulls during the course of the experiment**

	**Number of birds positive/number of birds examined per day**											
	**2 dpi**		**4 dpi**		**5 dpi**		**6 dpi**		**7 dpi**		**12 dpi**	
**Tissue**	**HE** ^**1**^	**IHC** ^**2**^	**HE**	**IHC**	**HE**	**IHC**	**HE**	**IHC**	**HE**	**IHC**	**HE**	**IHC**
**Thymus**	0/4	2/4	0/5	0/5	0/2	0/2	0/2	0/2	0/1	0/1	0/2	0/2
**Adrenal**	1/4	1/4	0/5	0/5	0/2	0/2	1/2	1/2	0/1	0/1	0/2	0/2
**Lung/Trachea**	0/4	3/4	1/5	0/5	1/2	0/2	1/2	0/2	0/1	0/1	0/2	0/2
**Air sacs**	4/4	4/4	5/5	5/5	2/2	1/2	1/2	1/2	0/1	0/1	1/2	1/2
**Pancreas**	0/4	0/4	2/5	2/5	1/2	1/2	2/2	2/2	1/1	1/1	1/2	0/2
**CNS**	0/4	0/4	5/5	5/5	2/2	2/2	2/2	2/2	1/1	1/1	1/2	0/2

^1^HE: presence of histological lesions by examination of hematoxylin-and-eosin-stained tissue sections.

^2^IHC: presence of viral antigen by examination of tissue sections stained by immunohistochemical technique.

On day 4, all birds (#40, 41, 42, 43, 49) had airsacculitis and air sac granulomas similar to those on day 2. The trachea in one bird (#42) had many heterophils in the lumen. The pancreas in two birds (#40, 49) had multiple foci of necrotic acinar cells with scarce associated inflammatory cells (Figure 3C). The cerebrum in all birds (#40, 41, 42, 43, 49) had multiple randomly distributed foci of necrotic neurons, characterized by chromatolysis and satellitosis (Figure 3E). These foci of necrosis were associated with predominantly perivascular infiltration of inflammatory cells, consisting of variable proportions of lymphocytes, plasma cells, macrophages, and heterophils. The cerebellum of one bird (#49) and brainstem of another one (#43) had similar lesions as the cerebrum. Virus antigen expression was observed in epithelial cells (#42, 49) and unspecified inflammatory cells (#40, 41, 42, 43, 49) of the air sacs, in acinar cells (#40, 49) of the pancreas (Figure 3D), and in neurons, glial cells, and ependymal cells of the brain, localized to lesions in these tissues (Figure 3F). There also was virus antigen expression in ganglion cells of the peripheral nervous system in adrenal gland (#43) in absence of histological lesions.

On day 5, airsacculitis and air sac granulomas were seen in both birds (#46, 50) and mild heterophilic tracheitis in one of them (#50). The myocardium of both birds showed scattered foci of mixed inflammatory infiltrate. The pancreas of one bird (#50) showed multifocal pancreatitis. The CNS of both birds, similarly to day 4 dpi, presented multifocal mixed encephalitis; however in one of them (#46) no lesions were observed in cerebellum. Viral antigen expression was observed in few inflammatory cells in air sacs and pancreatic acinar cells in bird #50, in nuclei of heart myocytes of bird #46, and in neurons, glial cells and ependymocytes in both birds (#46, 50), localized to lesions in these tissues. There also was virus antigen expression in ganglion cells of the peripheral nervous system in adrenal gland (#46) in absence of histological lesions.

On day 6, the air sacs presented scattered granulomas in one bird (#44), and mild tracheitis and multifocal adrenocortical necrosis could be seen in the other one (#45). The pancreas of both birds (#44, 45) showed multifocal pancreatitis. The CNS of both birds presented multifocal mixed encephalitis, similarly to the previous days; however, in one of them (#44), no lesions were observed in cerebellum. Viral antigen expression was observed in few inflammatory cells associated to air sac, adrenocortical cells, pancreas acinar cells, neurons, glial cells and ependymocytes, localized to lesions in these tissues.

On day 7 the only bird evaluated (#48) showed mild tracheal epithelial hyperplasia, and some granulomas in lungs and air sacs associated with *Aspergillus*-like hyphae. Kidney presented focal interstitital nephitis, tubular casts and gout and liver showed isolated hepatocyte death, activation of Kupffer cells. In the large and small intestine we observed a mild, diffuse and generalized lymphocytic enteritis; and the presence of a heterophilic granuloma in the cloaca. The pancreas showed multifocal pancreatitis. In the CNS, cerebrum and brainstem presented multifocal mixed encephalitis, while cerebellum showed no relevant lesions except choroiditis in the fourth ventricle. Viral antigen expression was observed in pancreatic acinar cells, neurons, glial cells and ependymocytes, localized to lesions in these tissues. There also was virus antigen expression sporadically in Kupffer cells in absence of histological lesions.

**Figure 4:**
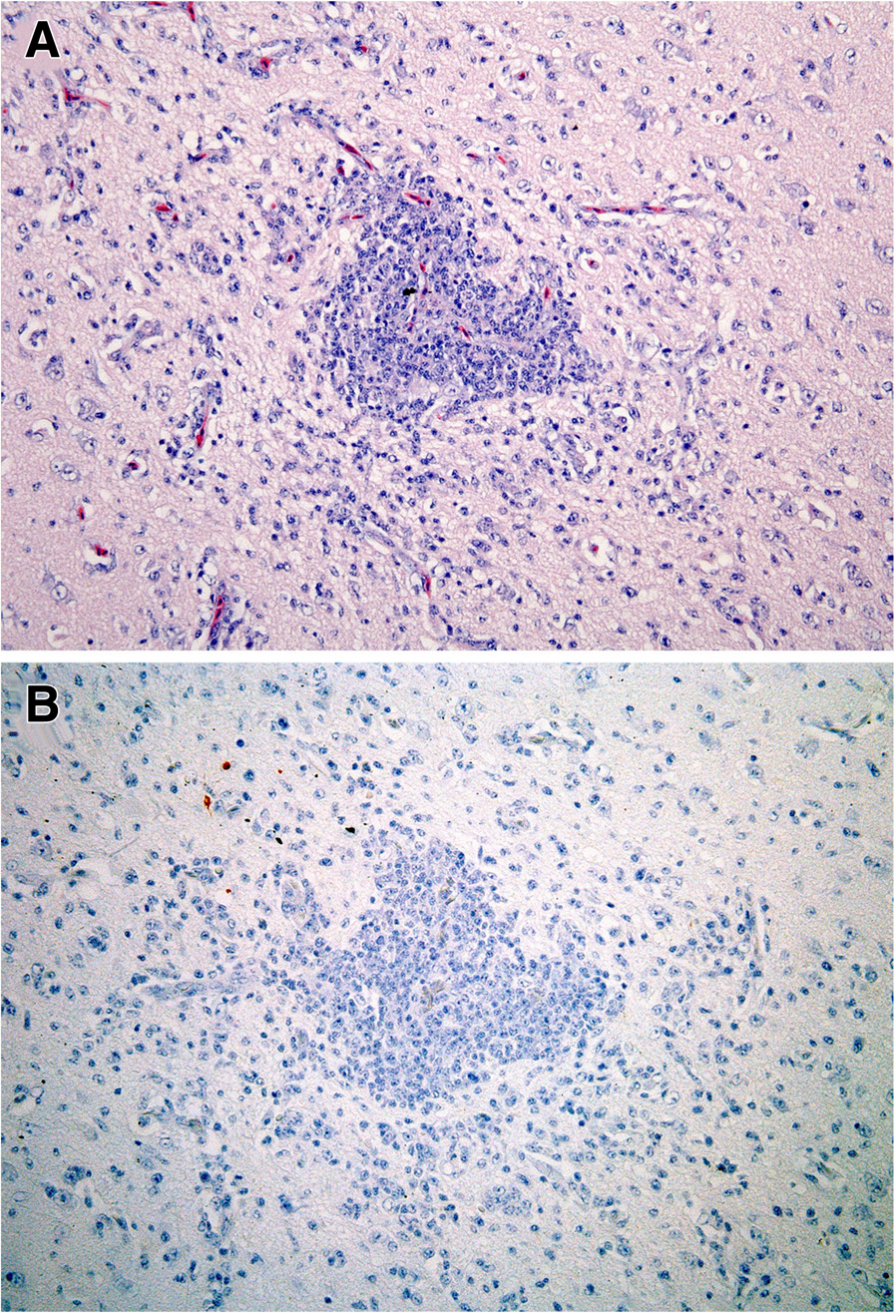
**Histopathology and antigen expression in central nervous system of gulls at 12 dpi infection.** By immunohistochemistry, influenza virus antigen expression is visible as a red staining. **(A)** Cerebral cortex showing intense perivascular cuffing and diffusely necrotic neurons and satellitosis. **(B)** Consecutive section of the same tissue without viral antigen expression. HE stain **(A)** and immunoperoxidase counterstained with hematoxylin **(B)**. Both the pictures are at 20× power fields.

On day 12, one infected bird (#51) showed airsacculitis and multiple granulomas consisting of a large central necrotic area containing bacterial colonies and a wide ring of inflammatory cells (histiocytes and heterophils). The other one (#47) showed mild scattered multifocal lymphocytic infiltrate in myocardium and pancreas; and severe inflammation in the CNS. Cerebrum, cerebellum and brainstem presented a severe multifocal mixed encephalitis (Figure 4A) and a severe lymphocytic choroiditis in lateral ventricles. Weak vestigial viral antigen expression was observed only in bird #51, located in epithelial cells of air sacs and in inflammatory cells of one of the air sac granulomas. Bird #47 showed no positivity at any organ or tissue (Figure 4B).

Tissues of sham-inoculated birds were examined according to the same methods as those of H5N1-inoculated birds. Histological lesions and viral antigen expression were not observed in sham-inoculated birds on days 3 and 12 dpi.

### Virus titration in selected tissues

Virus was isolated from tissues of all H5N1-inoculated birds euthanized or found dead between 2 and 7 dpi, but not at 12 dpi (Additional file 1). Virus was isolated from multiple organ and systems: respiratory, digestive, urinary, and lymphoid systems of all birds; brain in all but four birds; skeletal muscle in four birds; and skin in none of the birds. At 2 dpi, the highest viral titres (> 10^7^ TCID_50_ per g tissue) in each bird were observed in respiratory tract: bronchus or air sac. At 4 dpi, the highest viral titre in two of four birds was detected in the brain (≥ 10^7^ TCID_50_ per g tissue) whereas viral titres in respiratory tract tissues (trachea, bronchus lung, air sac) were lower than at 2 dpi (range: 10^2.7^- 10^6.4^ TCID_50_ per g tissue). From 5 to 7 dpi the highest viral titres were detected in brain and pancreas, and all the birds on those days showed a titre ≥ 10^7^ TCID_50_ per g tissue in one or both of these organs. Virus was not isolated in the tissues coming from sham-inoculated birds.

## Discussion

Our study shows that black-headed gulls are highly susceptible to disease from HPAIV A/turkey/Turkey 1/2005 (H5N1). All inoculated birds were productively infected and shed infectious virus until 7 dpi from the pharynx and 6 dpi from the cloaca, at maximal titres of more than 10^4^ TCID_50_ per mL. They developed systemic disease with a high morbidity and mortality rate and conspicuous nervous signs. The mortality was high and surviving animals showed mild nervous signs until the last day of the experiment. Infected birds presented pharyngeal and cloacal excretion detected by RT-PCR until the last day of the experiment, and viral isolation could be done until 6 dpi and 7 dpi, respectively, from pharyngeal and cloacal swabs (Table 2; Figure 1A, B). The results from these black-headed gulls can be compared directly with an earlier study on six wild duck species [10], because the virus, inoculation dose, and route of inoculation used were identical. The black-headed gulls had a slightly higher pattern of virus excretion to that of mallards (*Anas platyrhynchos*), Eurasian pochards (*Aythya ferina*), and tufted ducks (*A. fuligula*), which had a maximum titre of about 10^3^ TCID_50_ per mL and excreted up to 6 dpi. The black-headed gulls also were more susceptible to disease from HPAIV H5N1 than any of the six duck species studied. For the most susceptible duck species, the tufted duck, clinical signs were severe in 3 of 7 (43%) and mild in 4 of 7 (57%). In contrast, clinical signs were severe in 14 of 16 (87.5%) of H5N1-inoculated black-headed gulls and mild in 2 of 16 (12.5%). Together, these results indicate that black-headed gulls are more likely to act as sentinels of the presence of HPAIV H5N1 in the environment than as long-distance carriers of the virus to new geographical areas. Older black-headed gulls may be more resistant to disease from HPAIV H5N1 infection, since an age-related association with dissemination and clinical outcome in Peking ducks has been observed following infection with H5N1 HPAIV [18].

In our black-headed gulls, the clinical picture—showing exclusively neurological signs—was similar to that described for laughing gulls [6] and herring gulls [5]. However, none of our black-headed gulls showed total recovery, while some herring gulls recovered completely after experimental infection using two different HPAIV H5N1 isolates (A/Whooper Swan/Mongolia/244/05 and A/Duck Meat/Anyang/01), and one laughing gull, infected with HPAIV H5N1 (A/Whooper Swan/Mongolia/244/05 H5N1) recovered without clinical signs. In these species, clinically healthy birds showed pharyngeal viral excretion detected by viral isolation, but no longer than 10 dpi for laughing gulls [6] and 5 dpi for herring gulls [5]. Although comparison between our study and these studies is difficult because of differences in virus strains, inoculation dose, and route of inoculation, it suggests that black-headed gulls are more susceptible to disease from HPAIV H5N1 than either herring gulls or laughing gulls and show a similar level of virus excretion as laughing gulls. In the case of common gulls, it is not clear whether they are resistant to the infection with (A/Gull/Chany/P/06) H5N1 or this isolate is not pathogenic for gulls [7].

The absence of gross lesions in our black-headed gulls, despite abundant virus replication and associated histological lesions in multiple organs, is remarkable, but corresponds to results of other experimental HPAIV H5N1 infections in wild birds, where only a small proportion of infected individuals shows gross lesions [10]. Specifically, we did not observe multifocal pancreatic necrosis, the most common gross lesion in most wild birds, or multi-organ haemorrhage, as is seen specifically in swans [19]. We did observe a clear decrease of the weight in the H5N1-inoculated birds in comparison with sham-inoculated ones, and also between the birds that died and birds that survived within the inoculated group. These findings correspond with severe anorexia, leading to rapid loss in body weight.

**Figure 5:**
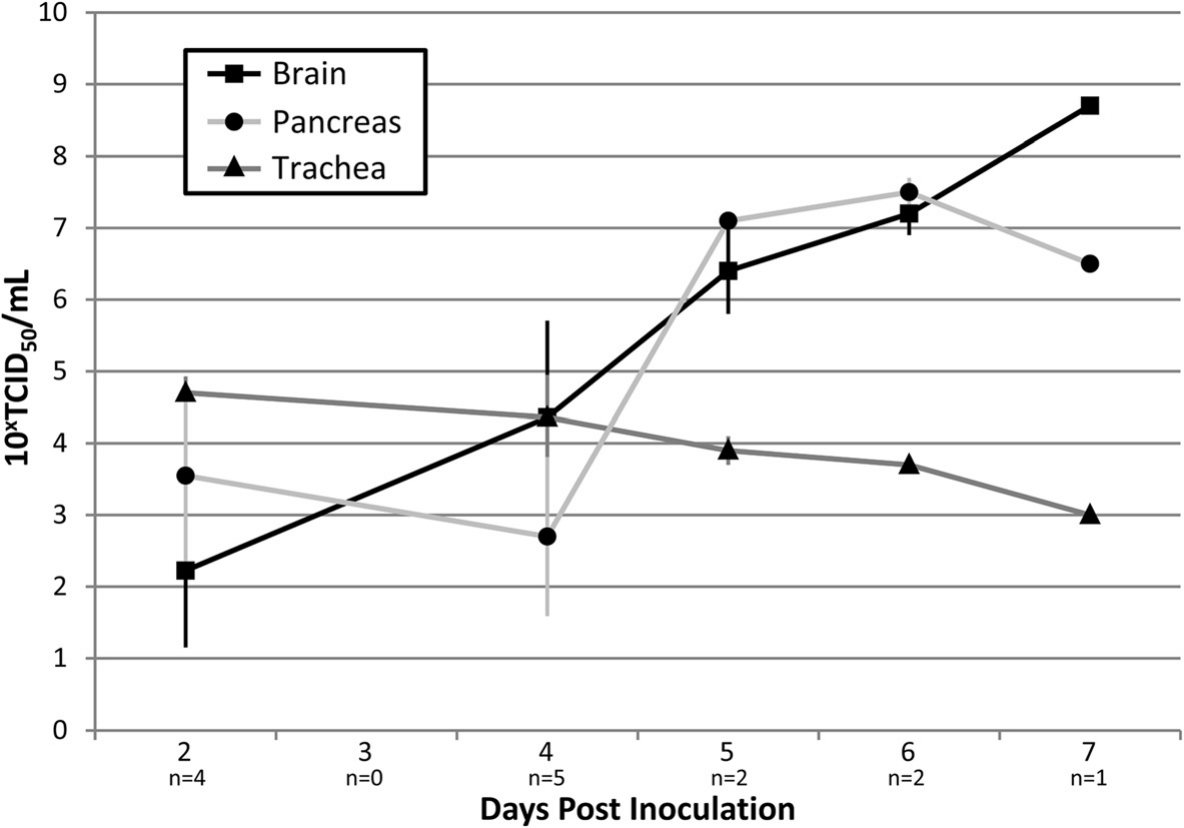
**Comparison of viral loads in selected tissues during the course of the experiment.** The viral loads are expressed as 10^x^ × median tissue culture infectious dose per mL (TCID_50_/mL), among trachea, brain, and pancreas in H5N1-inoculated black-headed gulls from day 2 to 7 post inoculation. The sample sizes (n) indicate the number of black-headed gulls that contributed to each mean value, with their corresponding standard errors (vertical bars).

The location and severity of microscopic lesions generally corresponded to the distribution and intensity of the immunostaining and to the viral load in each evaluated organ or tissue. At 2 dpi, viral antigen expression and viral titres were higher in organs of the respiratory tract than in other organ systems, although viral antigen was expressed in some other organs (thymus, adrenal gland). This suggests that the viremic period is reached very soon after virus inoculation. At 4 dpi, the respiratory tract presented a similar degree of microscopic lesions, viral antigen expression and viral titres as at 2 dpi. Moreover, necrotizing pancreatitis and neuronal necrosis in the brain were present, as described in other gull species (6, 5). Viral titres in pancreas and brain were similar to those respiratory tract tissues. From 4 dpi onwards, the severity of microscopic lesions, viral antigen expression and viral titres were increased in brain and pancreas and decreased in respiratory tract tissues. Interestingly, while the two birds euthanatized on day 12 dpi both showed neurological signs, and one of them showed microscopic brain lesions, no virus could be detected in the brain or any other tissue by virus isolation or immunohistochemistry in either bird (Table 3 and Additional file 1). As a general trend in this HPAIV H5N1 infection of black-headed gulls, we can assert that the virus is mainly in the respiratory tract on the first days after inoculation and then concentrates more in pancreas and CNS from 4 dpi onwards (Figure 5).

## Additional file

Additional file 1:
**Distribution of infectious virus in tissues of H5N1-inoculated gulls during the course of the experiment.**
