# Supplementary material for: Experimental infection of highly pathogenic avian influenza virus H5N1 in black-headed gulls (Chroicocephalus ridibundus)
Source: Vet Res. 2014 Aug 19;45(1):84. doi: 10.1186/s13567-014-0084-9 (PMC4189156; doi:10.1186/s13567-014-0084-9)
Supplement: Additional file 1: — Distribution of infectious virus in tissues of H5N1-inoculated gulls during the course of the experiment. [file 13567_2014_84_MOESM1_ESM.docx]

**Additional file 1** Distribution of infectious virus in tissues of H5N1-inoculated gulls during the course of the experiment.

|  |  | **Virus titre (log median tissue infectious dose per mL) per tissue of individual birds** | | | | | | | | | | | | | | | | | | | | | |
| --- | --- | --- | --- | --- | --- | --- | --- | --- | --- | --- | --- | --- | --- | --- | --- | --- | --- | --- | --- | --- | --- | --- | --- |
| **Day of necropsy** |  | **2 dpi** | | | |  | **4 dpi** | | | | |  | **5 dpi** | |  | **6 dpi** | |  | **7 dpi** |  |  | **12 dpi** | |
| **Bird ID.** |  | 36 | 37 | 38 | 39 |  | 40 | 41 | 42 | 43 | 49* |  | 46* | 50* |  | 44 | 45 |  | 48* |  |  | 47 | 51 |
| **Tissues** |  |  |  |  |  |  |  |  |  |  |  |  |  |  |  |  |  |  |  |  |  |  |  |
| Skin |  | - | - | - | - |  | - | - | - | - | - |  | NA | - |  | - | - |  | - |  |  | NA | - |
| Brain |  | 2.9 | 4.9 | - | 1.1 |  | 7.4 | - | 4.0 | 3.4 | 7.0 |  | 7.0 | 5.8 |  | 7.5 | 6.9 |  | 8.7 |  |  | - | - |
| Trachea |  | 4.6 | 4.7 | 5.3 | 4.2 |  | 2.8 | 5.2 | 5.0 | 3.1 | 5.7 |  | 3.7 | 4.1 |  | 3.7 | 3.7 |  | 3.0 |  |  | - | - |
| Bronchus |  | 7.1 | 7.0 | - | 6.1 |  | 4.8 | 4.9 | 6.4 | 3.8 | 4.9 |  | 5.5 | 6.1 |  | 4.2 | 5.3 |  | 5.5 |  |  | NA | - |
| Lung |  | 6.9 | 4.8 | 3.8 | 6.0 |  | 3.4 | 3.8 | 3.8 | 2.7 | 4.5 |  | 5.5 | 5.9 |  | 5.8 | 6.7 |  | 4.6 |  |  | NA | - |
| Air sac |  | 6.6 | 7.2 | 6.2 | 7.5 |  | 3.2 | 5.1 | 5.4 | 5.7 | 5.1 |  | 5.2 | 5.3 |  | 5 | 5.7 |  | - |  |  | - | - |
| Thymus |  | 3.7 | 5.8 | - | 2.7 |  | - | - | 3.5 | - | - |  | - | 2.8 |  | 3.3 | - |  | - |  |  | - | - |
| Proventriculus |  | 5.9 | 4.5 | 3.9 | 4.5 |  | 3.2 | 4.1 | 4.7 | - | 3.8 |  | 3.5 | 4.8 |  | 4.5 | 5.3 |  | 2.9 |  |  | - | - |
| Duodenum |  | 5.6 | 5.2 | - | 4.7 |  | - | 2.8 | 4.5 | 2.3 | 2.1 |  | 3.9 | 3.9 |  | 5.1 | 4.9 |  | 2.8 |  |  | - | - |
| Pancreas |  | 4.2 | 5.4 | - | 4.6 |  | - | 4.0 | 4.7 | - | 4.8 |  | 7.1 | 7.1 |  | 7.7 | 7.3 |  | 6.5 |  |  | - | - |
| Jejunum |  | 4.4 | 4.8 | 4.0 | 4.7 |  | - | 4.6 | 3.6 | - | 3.0 |  | 4.5 | - |  | 4.6 | 4.6 |  | 3.5 |  |  | - | - |
| Colon |  | 6.0 | 5.1 | 3.2 | 4.7 |  | 2.6 | 2.4 | 3.8 | 2.2 | 3.7 |  | 5.1 | 2.6 |  | - | 3.5 |  | - |  |  | - | - |
| Bursa of Fabricius |  | 4.9 | 5.6 | 3.6 | 4.2 |  | 3.4 | 4.2 | 2.6 | 1.9 | NA |  | 5.3 | 3.6 |  | 2.5 | 3.8 |  | 5.0 |  |  | - | - |
| Spleen |  | 4.6 | 5.3 | 5.9 | 3.7 |  | 3.8 | 5.2 | 5.3 | 2.9 | 3.5 |  | - | 2.2 |  | 1.9 | 3.0 |  | 2.5 |  |  | - | - |
| Liver-Gallbladder |  | 4.1 | 3.6 | 3.6 | 3.2 |  | 3.2 | 2.4 | 2.1 | - | 2.5 |  | 2.4 | 2.7 |  | 3.9 | 2.4 |  | - |  |  | - | - |
| Kidney |  | 4.6 | 5.9 | 3.6 | 3.8 |  | 3.5 | 4.8 | 4.8 | 4.5 | 3.6 |  | 5.9 | 4.4 |  | 3.5 | 4.6 |  | 2.9 |  |  | NA | - |
| Skeletal muscle |  | - | 2.7 | - | - |  | 2.0 | - | - | - | - |  | - | 1.6 |  | 1.7 | - |  | - |  |  | - | - |
|  |  |  |  |  |  |  |  |  |  |  |  |  |  |  |  |  |  |  |  |  |  |  |  |

* Birds that died spontaneously. Pharyngeal and cloacal viral shedding in these birds were respectively: 3.5/<0.5 (#49), 2.5/<0.5 (#46), 3.2/<0.5 (#50), 1.5/<0.5 (#48).

NA: not Available.

- , no virus isolated; yellow, virus titre 1.1-2.9; green, 3.0-4.9; red, 5.0-6.9; brown, 7.0-8.7.
